# The Impact of the Highly Virulent SARS-CoV-2 Gamma Variant on Young Adults in the State of São Paulo: Was It Inevitable?

**DOI:** 10.7759/cureus.26486

**Published:** 2022-07-01

**Authors:** Beny Spira

**Affiliations:** 1 Microbiology, Universidade de São Paulo, São Paulo, BRA

**Keywords:** selection, mutations, γ, voc, sars-cov-2, covid-19, pandemic

## Abstract

Background

The coronavirus disease 2019 (COVID-19) pandemic had and is still having a tremendous impact on people all over the world, but it has been particularly harsh in South America. Nine out of 13 South American countries are among the 50 countries with the highest COVID-19 death rates. The gamma severe acute respiratory syndrome coronavirus 2 (SARS-CoV-2) variant that emerged by the end of 2020 in the Brazilian Amazon quickly spread throughout the country causing the harsh COVID-19 second wave. This variant displayed high viral loads, high transmissibility, and increased virulence as compared to previous variants.

Aims

The aim of this retrospective study is to revisit and analyse the epidemiology of the COVID-19 second wave in the state of São Paulo, the most populous Brazilian state. In addition to examining the possible factors that led to the emergence and propagation of the gamma variant, measures that could have prevented its spread and that of other highly virulent variants were also investigated.

Materials and methods

Data from São Paulo's official sources on morbidity, mortality, age distribution, and testing prior to and during the COVID-19 second wave (February - June 2021) and data regarding the distribution of SARS-CoV-2 variants in the country were parsed, analyzed, and compared to the period that anteceded the eruption of the second COVID-19 wave.

Results

In the state of São Paulo, the toll of the COVID-19 second wave surpassed that of the first 11 months of the pandemic (from March 2020 to January 2021), as 56% of the deaths occurred in the five months of the second wave between February and June 2021. The mean age of COVID-19 victims, which was already below life expectancy in the state dropped even further in the pandemic's second wave, reaching an average of 60 years of age. The years of life lost per death per month doubled and the case-fatality rate (CFR) of young adults (20-39 years old) more than trebled during this period. A number of hypotheses have been raised that might explain the emergence and spread of the gamma variant and the measures that could have been taken to prevent it and minimise its impact on the population.

Conclusions

Over 142,000 people died as a result of the SARS-CoV-2 gamma variant sweep in São Paulo in the first semester of 2021. Due to its high viral load, the gamma variant displayed high transmissibility and a high degree of virulence resulting in increased case fatality rates across most age tiers. Notably, this second wave was marked by a very significant increase in deaths among young adults. This increase was at least partially due to a deterioration in general health provoked by non-pharmaceutical interventions. In hindsight, a safer and more effective measure might have been to allow the free spread of the virus among the young and healthy in the first wave, thus conferring immunity against more virulent variants that emerged later on.

## Introduction

The aetiological agent of coronavirus disease 2019 (COVID-19), severe acute respiratory syndrome coronavirus 2 (SARS-CoV-2), rapidly swept across the world reaching all countries in all inhabited continents, resulting in a global pandemic of high proportions [1]. The COVID-19 pandemic reached Brazil by March 2020, causing the first wave of infections and thousands of cases, deaths, mobility constraints and other social restrictions. The COVID-19 second wave erupted in December 2020 in the state of Amazonas and soon after that in other parts of Brazil. To date (March 2022), more than 650,000 Brazilians perished due to COVID-19 and 30 million had confirmed infections [2].

SARS-CoV-2 evolves with a mutation rate of about $3 \times 10^{-6}$ mutations per nucleotide per replication cycle, in line with other coronaviruses [3]. This translates to $\sim 0.5$ mutations per infected individual if we assume a maximum limit of $3 \times 10^8$ infectious units produced over the course of an infection [3]. With such a relatively high mutation rate, the emergence of many new variants is expected in the course of a pandemic, however, only a few of them confer some significant selective advantage to the virus and effectively increase their share in the population pool. Some of these new variants are of public health concern (known as ’variants of concern’ or VOC) either because they have an increased capacity of causing severe illness or because they are more efficiently transmitted than other variants. Different VOCs appeared in several parts of the world, for instance, the alpha VOC (B.1.1.7) emerged in the United Kingdom [4]; beta (B.1.351) appeared first in South Africa [5]; gamma (P.1 or B.1.1.28) emerged in Brazil [6]; delta (B.1.617) was first detected in India [7] and the omicron VOC emerged in South Africa in November 2021. All of these VOCs carry mutations that are associated with increased transmission and/or disease severity. Some of them also display higher lethality and immune escape [8].

The gamma VOC was first detected in Manaus by November 2020 [6] where it caused in the subsequent months a severe COVID-19 second wave with a record number of cases and deaths in the Amazonas state [9]. From there, the gamma variant disseminated throughout the country with a similar pattern of high lethality. The gamma variant differed from its most closely related ancestral by a total of 25 mutations (synonymous and non-synonymous), including 10 amino acid substitutions in the spike protein, some of them associated with a higher degree of transmissibility [6,10]. In addition, the viral load in patients infected with the gamma variant was 10-fold higher than in those with other SARS-CoV-2 variants [11]. These features likely contributed to the increased virulence (defined as virus-induced host mortality [12]) of this new variant. It has been claimed that part of the devastating effects of the gamma variant in Manaus could be ascribed to the already debilitated health care system and unpreparedness of the local health authorities to the new COVID-19 wave [13]. Nonetheless, the upsurge in cases and deaths did not remain restricted to Manaus and bordering counties but rapidly swept through most Brazilian urban centres [14-23]. A glimpse of the literature regarding the clinical and epidemiological patterns of the gamma variant throughout Brazil reveals similar characteristics. For instance, Oliveira et al. reported that young patients aged 20-29 years old diagnosed in February 2021 in the southern state of Paraná displayed a three-fold higher risk of death compared to those diagnosed in January 2021 [19]. The case-fatality rate (CFR) in this age group went from 0.04% in January (before the arrival of the gamma variant) to 0.13% in February while patients from 30 to 59 years old showed approximately a doubled CFR. Similarly, in the Rio Grande do Sul state (also in southern Brazil), the proportion of COVID-19 deaths among patients under the age of 60 increased from 18% in November and December to 28% in February [20]. Not only were the COVID-19 victims younger than before, but they were also healthier, as the share of patient deaths without pre-existing risk conditions increased from 13% to 22% in the second wave [20].

## Materials and methods

Epidemiological analysis

The progression of the COVID-19 pandemic in the state of São Paulo was followed from March 2020 to June 2021, encompassing two major waves. Data on morbidity, comorbidity and mortality were retrieved from Seade [24] on 29.11.21 and parsed with the help of the awk programming language. CFR was calculated by dividing the number of deaths accumulated in each month by the number of confirmed cases accumulated in the same month. "Years of life lost/number of deaths" were calculated as follows: $\sum \frac{(76 - A) }{D}$, where *76* is the life expectancy in the state of São Paulo, *A* is the age of death of each victim and *D* is the number of monthly deaths.

SARS-CoV-2 variant data

Data about variant prevalence in Brazil were downloaded from the GISAID database (http://www.gisaid.org) on 31.10.21.

Statistical analysis

Statistical differences in the mean age of death were evaluated by analysis of variance (ANOVA) followed by Tukey’s post hoc analysis with the JASP software [25].

## Results

COVID-19 second wave in the state of São Paulo

An overview of the epidemiology of the second wave of COVID-19 in São Paulo, Brazil, whose critical period spanned February to June of 2021 is presented below. Similarly to what has occurred in the great Spanish flu pandemic, the COVID-19 second wave was considerably harsher than the first one. In the state of São Paulo, as in other Brazilian states, the gamma SARS-CoV-2 variant was remarkably deadlier and more transmissible than the original Wuhan lineage and other variants that circulated during the first wave and in the subsequent months prior to the emergence of the gamma VOC, particularly toward patients belonging to the 20-59 age group.

**Figure 1:**
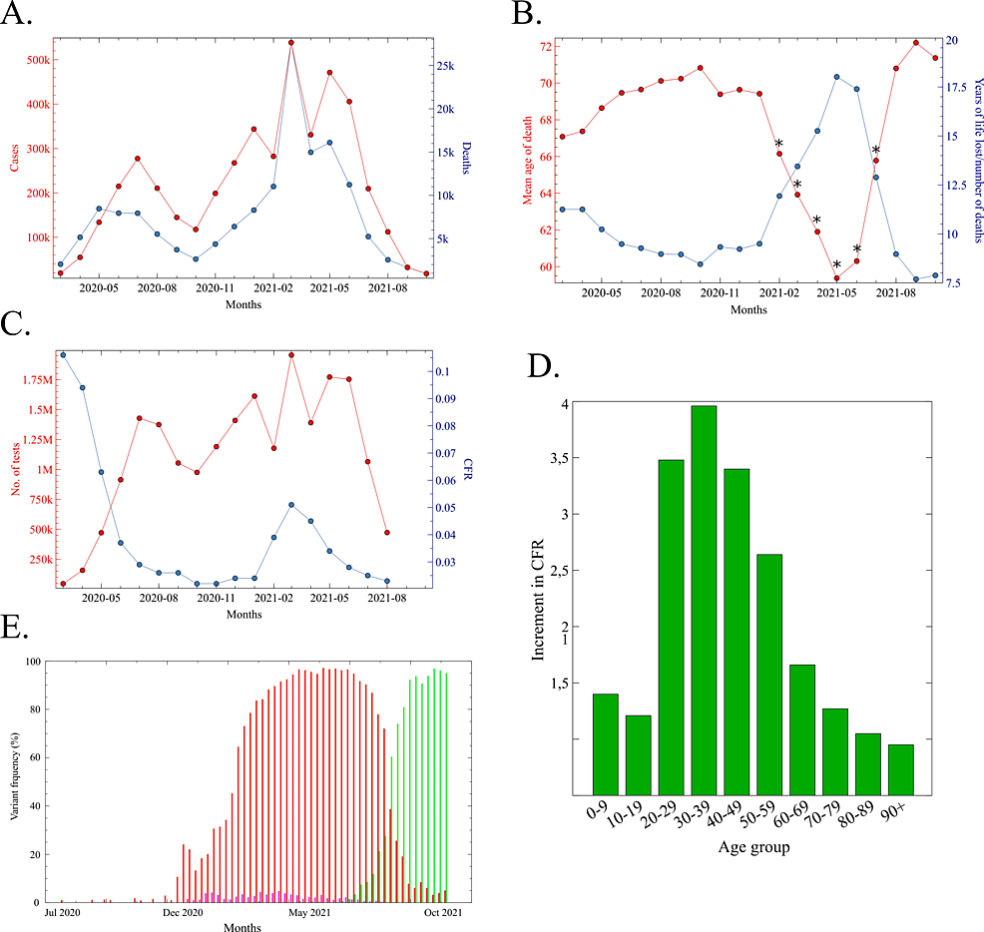
Epidemiological data of COVID-19 in the state of São Paulo A. COVID-19 cases (red) and deaths (blue) in the state of São Paulo throughout the pandemic, from March 2020 to October 2021. B. The average age of COVID-19 deaths (red) and years of life lost per number of deaths in each month (blue) throughout the pandemic (March 2020 - October 2021) in the state of São Paulo. Asterisks represent statistically significant differences between the mean age of death in each month from February to July 2021 and the mean age of death in all other months ($p&lt;0.001$; ANOVA followed by post hoc Tukey analysis). C. The number of COVID-19 tests per month (red) and the evolution of the CFR (blue) in the state of São Paulo. D. CFR increase in the different age groups in the COVID-19 second wave in the state of São Paulo. The mean CFR of each age group in the Feb-Jun 2021 period was divided by the corresponding mean CFR of each age group in the Jul-Dec 2020 period. E. Frequency of SARS-CoV-2 VOCs alpha, beta, gamma and delta in Brazil (from July 2020 to October 2021). Total number of sequenced VOCs = 59,982; total number of sequenced genomes = 64,378. Red bars, gamma; green bars, delta; pink bars, alpha; blue bars, beta (almost invisible due to very low frequency). Plots shown in A, B, C, and D were calculated from data downloaded on 29.11.21 from Seade [24]. Testing data in C was downloaded from https://www.saopaulo.sp.gov.br/planosp/simi/dados-abertos/ and VOCs frequency in E was downloaded on 31.10.21 from http://www.gisaid.org. ANOVA: analysis of variance; CFR: case-fatality rate

From March 2020 to June 2021 (16 months in total), approximately 4 million COVID-19 cases and over 142,000 COVID-19 deaths were registered in the state of São Paulo [24]. Of these, 50.3% of cases and 56.2% of deaths occurred during the five months of the second wave (February-June 2021) (Figure 1A).

In line with low- and middle-income countries [26-27], the average age of COVID-19 deaths in São Paulo was below the life expectancy at birth (76.4 years) [28]. Prior to February 2021, the average age of COVID-19 victims in the state was 69.3 years, plunging hereafter and reaching 59.4 years by May 2021 (Figure 1B). Accordingly, the average years of life lost per month was, before February 2021, 9.63 years/death/month; this number increased to 18 years/death/month at the height of the 2nd wave (Figure 1B). Following the retreat of the gamma variant (Figure 1E), the mean age of COVID-19 deaths went up to 71-72 years and the years of life lost declined to around 7.5 per death per month. These data show that the gamma VOC not only was overall more lethal than previous variants but that it was considerably deadlier towards younger people.

COVID-19 CFR oscillated throughout the pandemic (Figure 1C). It was artificially high in the first three to four months (March to June 2020) due to the low level of testing (Figure 1C). As the number of tests increased, the CFR declined, stabilising at under 3% by July 2020 and maintaining this rate until February 2021, the month that marks the beginning of the steep increase of deaths in the second wave. Henceforth, the CFR increased by more than two-fold, indicating the spread of the virulent gamma variant, as had been previously observed in Manaus [20]. The number of tests was reduced considerably after July, and soon after, the government of São Paulo ceased publishing data about testing implementation.

A break by age group shows that the impact of the gamma variant on the CFR was not evenly distributed among the different age categories. While the CFR of the youngest (0-19) and the oldest (60-90+) changed little or not at all in the second wave, the CFR of young adults (20-59 years) increased 2.6 to four-fold in this same period (Figure 1D). The lack of impact on the mortality of the 60-90+ population could not be ascribed to vaccination, as mass vaccination of > 70-year-old people began only on 15 March [29-30], when the second COVID-19 wave was already ravaging the state (Figure 1A). These data reinforce the evidence that the gamma variant was particularly deadly toward young adults.

The highly virulent gamma variant quickly swept the country such that by May 2021 > 90% of the sequenced genomes in Brazil belonged to the gamma lineage (Figure 1E). The gamma variant predominated in Brazil until the end of July 2021. From then on, it was rapidly replaced by the delta VOC, by mid-September, only 4% of all SARS-CoV-2 sequences in Brazil corresponded to gamma.

The impact of the gamma VOC on young adults

Because young adults were more severely burdened by the COVID-19 second wave than other age tiers, from now on, we are going to focus on the 20-39 years age group. The 20-39 age group represents 32.2% of São Paulo’s population. From March 2020 to January 2021, people in this age group constituted 41.4% of the registered COVID-19 cases but only 3.6% of the deaths. In the months that followed (February-June 2021) the share of cases of this age group remained almost unaltered (39.6%), but their proportion in the pool of deaths rose to 7.5%. Accordingly, the CFR of the 20-39 tier climbed from 0.2% (July-December 2020 average) to 0.75% (February-June 2021 average), a 3.75-fold increase (Figure 1D). Seventy-three per cent of the deaths in the 20-39 age group occurred in the Feb-Jun 2021 period. In addition, while 68% of the 20-39 years COVID-19 victims up to January 2021 carried at least one underlying health issue, this proportion dropped to 53% in the second wave (Table 1). Thus, during this five-month period, the profile of COVID-19 deaths changed considerably, with a shift to younger and healthier victims, confirming what has been reported by other clinical and epidemiological studies regarding the increased virulence of the gamma VOC in young adults [14-23].

**Table 1:** The impact of COVID-19 on the 20-39 age group in terms of cases and deaths from March 2020 to June 2021 ^1 ^relative to the total number of cases ^2^ relative to the total number of deaths ^3^ relative to the number of deaths in Mar 2020 - Jan 2021 ^4^ relative to the number of deaths in Feb - Jun 2021 Data downloaded from Seade [24] on 29.11.21.

	Cases or Deaths	% of the Total
Cases (total)	1615906	-
Deaths (total)	8214	-
Cases (Mar 2020 - Jan 2021)	820635	50.8^1^
Deaths in (Mar 2020 - Jan 2021)	2248	27.4^2^
Deaths (Mar 2020 - Jan 2021) with at least one comorbidity	1533	68.2^3^
Deaths (Mar 2020 - Jan 2021) with zero comorbidity	715	31.8^3^
Cases (Feb - Jun 2021)	795271	49.2^1^
Deaths (Feb - Jun 2021)	5966	72.6^2^
Deaths (Feb - Jun 2021) with at least one comorbidity	3151	52.8^4^
Deaths (Feb - Jun 2021) with zero comorbidity	2815	47.2^4^

It is possible that the elevated mortality observed in São Paulo during the second wave was a byproduct of the chaotic healthcare response to hospital overload. However, while the increase in hospital occupancy may have contributed to exacerbating the mortality rate in some cases, the vast majority of deaths were not caused by hospital overload for a couple of reasons. First, even at the peak of the second wave, the proportion of used ICU beds in the state of São Paulo never reached 100% [31]. Second, the CFR did not increase in all age groups (Figure 1D) as was expected if the cause of the CFR increase was poor healthcare response. We may thus conclude that the high level of mortality observed during the COVID-19 second wave was primarily due to the emergence and spread of the highly lethal gamma variant.

## Discussion

Could the emergence and spread of the gamma variant have been prevented?

The selection and spread of the gamma variant had adverse consequences for almost all age groups, but its impact was particularly harsh on young adults (20-39 years), who had largely been spared before 2021. In the first 11 months of the COVID-19 epidemic in São Paulo (Mar 2020 - Jan 2021), there were 2248 COVID-19 deaths in the 20-39 age group, 31.8% of those (715) with no known comorbidity. In the five months that followed (February - June 2021), the number of COVID-19 victims in this age group was 5966, of which 2815 (47.2%) had no known comorbidity (Table 1). Had the CFR of this age group remained 0.2% (the average CFR before February 2021) throughout the 16 months of the pandemic, the expected number of deaths would have been 3232 ($1,615,906\ cases\ \times\ 0.2\%$) instead of the 8214 registered COVID-19 deaths. In other words, the outstanding increase in the CFR (3.75 times) of young adults, caused by the spread of the gamma variant, resulted in the death of an additional 4982 people in this age tier (60.7% of 8214), confirming what has been observed elsewhere [14-23]. In fact, the number of deaths would have been even lower had the gamma variant not emerged, as there would be fewer cases as well (2.13 times more cases/month were registered in the five-month second wave than in the previous 11 months). Assuming that the emergence and spread of the gamma variant were inevitable, prior exposure to other SARS-CoV-2 variants might have conferred immunity against the gamma variant to the vast majority of the 20-39 age group. This could have been done by not implementing the physical distancing measures in the state of São Paulo during the first 11 months of the epidemic. The problem with this proposition is that in theory, the lack of physical distance and other restrictions would have caused a considerable increase in the number of cases and a proportional increase in the number of deaths. But, would it? Ultimately, the number of deaths depends on the virus prevalence in a population. The proportion of the population that is immune to COVID-19 remains unknown. We include in this group both people that are genuinely immune (do not get infected by SARS-CoV-2) and those that are completely asymptomatic. It is very unlikely that 100% of the population is susceptible to COVID-19 for several reasons: (I) in February 2022, two years after the WHO declared the COVID-19 pandemic, the prevalence of cases reached only 13% of the Brazilian population; (II) a considerable share of the world population is likely to be naturally immune to COVID-19, as studies have reported that 20% to 50% of people not previously exposed to SARS-CoV-2 displayed T-cell reactivity to this virus [32-34]; (III) the disease is asymptomatic in many people, notably in children and teens [35-37]. Therefore, it is reasonable to argue that a high proportion of Brazil’s inhabitants was either immune or asymptomatic to COVID-19, at least until the emergence of the omicron variant.

The assessment of the COVID-19 infection-fatality rate (IFR) is notoriously difficult and attempts have been made to calculate its value with more or less success [38-41]. For instance, Irons and Raftery [41] estimated that the real number of infected people is likely to be at least twice as high as the official number of cases. However, some reports have claimed that extensive reverse transcription-polymerase chain reaction (RT-PCR) testing, as has been done in several places around the world, is a reliable tool to infer the number of infections in a population [39]. An assessment of the COVID-19 prevalence in Manaus in October 2020 (before the emergence of the gamma variant) based on an extrapolation of seroprevalence data came out with an incredibly low IFR based on an estimated attack rate of 76% [42], a proportion at or close to the herd immunity of Manaus’ population. Clearly, this number was an overestimation, given the explosion of the COVID-19 second wave two months later with twice more deaths than has been previously observed (2889 deaths from 01 Apr to 31 Oct 2020; 5659 deaths from 01 Dec 2020 to 30 Apr 2021 [2]). It has been claimed that the high numbers of cases and deaths in Manaus’ second wave could have been caused by reinfections [43], but no concrete evidence was provided in this regard. Other studies have shown that previous COVID-19 infection confers a high degree of immunity [44-45] and that reinfections, when they occur, have 90% lower odds of resulting in hospitalization or death than primary infections [46].

In light of the above, it is reasonable to estimate that the maximum proportion of the population that is susceptible to COVID-19 is likely to be between 20% and 25%, which means that at least 75% of the population is either immune or asymptomatic. Surely, this proportion varies among different countries and among different populations in a single country. Countries with young populations are expected to have lower levels of prevalence than countries with significant older populations. In São Paulo, the fraction of 20-39 years people with confirmed infections (cases) up to July 2021 was 11.2% (1,615,906 cases in a 14,390,429 population). Assuming that the upper limit of symptomatic infections is 25% of the population the corresponding maximum number of COVID-19 cases would have been 3,597,607. With a CFR of 0.2% (the CFR before the emergence of the gamma variant), 7195 deaths would be expected, 1019 fewer deaths than were actually registered until 30 June 2021. The impact on the 20-39 age group bearing no comorbidities would have been even more dramatic. The CFR in this subgroup increased 6.2 times - from 0.06% before gamma emergence to 0.37% in the second wave. With a CFR of 0.06%, the maximum number of deaths in this group during the entire pandemic would have been 40% lower, i.e., 2159 victims (0.06% of 3,597,607) instead of the 3,530 registered until the end of June 2021. It should be noticed that the predicted number of deaths in the absence of the gamma variant should have been even lower because in this back-of-the-envelope calculation, we did not take into account the mass (herd) immunity build-up by recovered people that would eventually protect the remaining susceptible young people from acquiring COVID-19 [47].

Some of the unintended consequences of the physical distancing measures, such as general health deterioration caused by diminished physical activity and weight gain may have contributed to increasing the proportion of obese individuals, important comorbidity that is associated with increased risk of hospitalization, critical care admission, and fatalities, especially in patients under 60 years [48-49]. In fact, it has been reported that the intake of highly caloric ultra-processed food increased [50], which along with a reduction in mobility and physical activities [51] resulted in heightened levels of obesity during the COVID-19 pandemic [52-55]. These trends were particularly pronounced among persons with already high BMI, which increased even further during the pandemic [55]. Indeed, the risks associated with COVID-19 are greatly enhanced as the patient’s BMI increases; for instance, a person with a BMI > 40 kg/m^2^ has a significantly worse prognosis than one with a BMI of about 30 kg/m^2^ [56]. As a result, a non-negligible fraction of young adults that perished in the pandemic second wave acquired an extra vulnerability caused by weight gain during the first year of the pandemic that may have ultimately contributed to their deaths.

Additionally, the restrictions imposed by the state might have affected people’s resilience against the gamma variant in other ways as well: (1) by preventing or diminishing immunity to the gamma variant, which might otherwise have been acquired through exposure to other coronaviruses or to less-lethal SARS-CoV-2 variants. Indeed, it is believed that the low COVID-19 death rate in Japan and other Pacific Rim countries can be partially ascribed to previous exposures to other coronaviruses, which might have conferred cross-protection against SARS-CoV-2 [57-60]; (2) lockdowns, curfews and the move to home-based work prevented direct exposure to sunlight, which is necessary for the biosynthesis of vitamin D from 7-dehydrocholesterol. Though the evidence in favour of vitamin D's role in COVID-19 prevention and treatment is still inconclusive [61], it has been shown that patients with severe vitamin D deficiency have a higher degree of hospitalisation and mortality risk [62-63]. Thus less exposure to sunlight due to mobility restrictions may have caused a reduction in vitamin D serum concentration, which, in turn, contributed to a severe outcome in some patients; (3) by limiting, disrupting or halting physical activity. It is known that physical exercises act as modulators of the immune system [64] and that moderate-intensity exercises are “immunoenhancing” [65-66]. The mobility restrictions, the move to home-based work and the forced closing of gyms have all contributed to a general decrease in physical activity, which might have otherwise helped the organism fight against respiratory infections such as COVID-19.

Historical analysis of the 1918 Spanish flu and other XX century flu pandemics shows that the second and subsequent pandemic waves were more severe than the first one [67]. Furthermore, data from military and civilian populations in the United States, UK, and Australia provide strong epidemiological evidence of cross-protection between the first and second 1918 flu pandemic waves [68]. It has been estimated that infection with influenza in the 1918 spring (first wave) provided a 35%-94% protection against the disease in the fall (second wave) and 56%-89% protection against death [68]. Similarly, a 61% protective effect against mortality was conferred by infection during the Spanish flu first wave in the autumn in the Australian army [69]. One lesson that could be drawn from these historical events is that exposure to the respiratory virus causing the first wave of a pandemic might confer cross-protection to subsequent waves and that this evidence should have been taken into account before implementing radical public health interventions designed to limit exposure to the virus [68]. This is even more true in the case of COVID-19 that, unlike the flu, was very selective in the first wave targeting mainly older and immunocompromised people. These particular groups could have been specifically protected, allowing the children, young and healthy to acquire immunity against the new virus, preventing unnecessary deaths and shortening the length of the pandemic [70-71].

Could the emergence of VOCs have been predicted?

With a mutation rate of $\sim 3 \times 10^{-6}$/nt/replication cycle ( $\sim 0.5$ mutation per infection and an evolutionary rate of $\sim 3$ mutations per month) [3], the emergence and selection of variants with an increased power of transmission were inevitable. The more important question is whether the emergence of more virulent variants could have been predicted. The answer to this question is less clear. On the one hand, high levels of virus replication, as ensued during a pandemic, can speed viral evolution simply by increasing genomic diversity and the chances of acquiring beneficial mutations. From this perspective, the most effective strategy to prevent the emergence of new and more virulent variants is by limiting the number of infections in the susceptible population as much as possible [72]. In addition, high levels of infection can increase the probability of coinfections, which may foment, through recombination and reassortment, the emergence of new and more virulent strains [73-74]. However, there is no evidence that the gamma variant evolved through recombination events. In fact, the gamma lineage, first detected in November 2020, harbours a total of 25 mutations (synonymous and non-synonymous) compared to its closest ancestral (isolated in March 2020) [6]. Given that SARS-CoV-2 evolves at a rate of three mutations per month [3], there was ample time for the gamma variant to acquire the relevant mutations without resorting to recombination.

**Figure 2:**
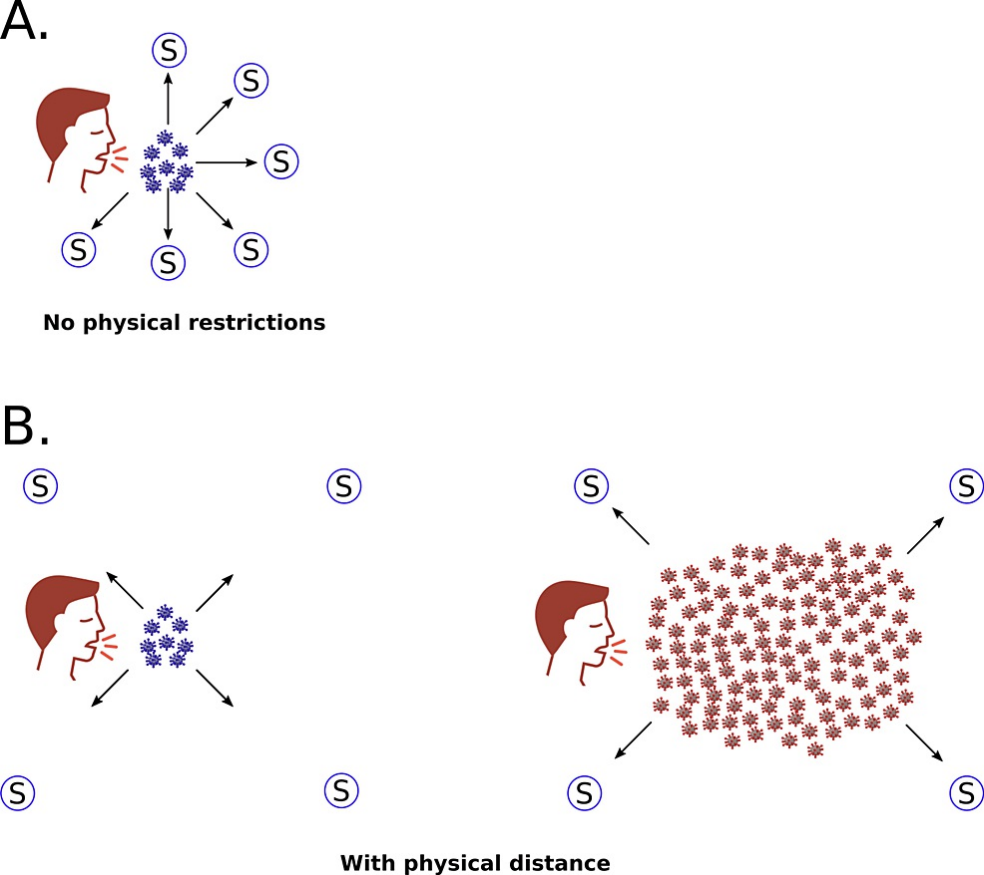
Schematic representation of the transmission dynamics of different SARS-CoV-2 variants (A) Spread of SARS-CoV-2 variants that produce low viral loads under conditions of unrestrictive physical contact; (B) spread of SARS-CoV-2 variants under physical distancing restrictions. Left: variants that produce low viral loads (and are, therefore, less virulent), cannot successfully spread throughout the host population. Right: a highly virulent/highly transmissible variant, such as gamma, produce high viral loads and has an advantage under restrictive physical distancing conditions. S represents potential susceptible hosts.

It is generally believed that the rate of pathogen transmission and virulence are positively associated with the degree to which the pathogen exploits its host and that both virulence and transmission rates are enhanced by within-host high replication rates [75-77]. For instance, in influenza, both transmission and virulence are affected by mutations that result in increased rates of pathogen replication in the host [78]. Likewise, several studies have shown a direct association between SARS-CoV-2 viral load, disease severity and mortality [79-85]. Higher viral loads enhance the rate of contagion as more virus particles are available to be transmitted by shedding or through other mechanisms (Figure 2). The notion that a high viral nasopharyngeal load increases viral transmission is indeed quite intuitive and was, in fact, confirmed by the data [86-90]. Thus, mutants that produce high viral loads in infected tissues, in particular, in the host’s upper respiratory tract have a selective advantage over competitors during the course of an epidemic. The selection of high-viral-load mutants is likely to be stronger under conditions that hinder free viral transmission such as abnormal physical distancing among the host population during an epidemic (Figure 2). In this case, variants that produce high viral loads would be also the ones with the highest ability of transmission and with the highest probability of fixation even at the cost of reducing the life span of the host. Though this hypothesis cannot be directly tested, there is now strong evidence that positive selection has played a pivotal role in the emergence and spread of the alpha, beta and gamma VOCs [10,91].

At least five convergent mutations emerged in those fast-spreading SARS-CoV-2 lineages where each of them, either alone or in combination, provides some significant fitness advantage [10]. Since natural selection occurs as a response to selective pressure, it has been proposed that the unnatural restrictions imposed on the host population provided the necessary selective pressure that eventually selected the gamma variant. Conversely, under conditions of exponential growth in a population of naive susceptible hosts there is no significant selective pressure on the pathogen, in this case, a slow mutational pattern guided by random genetic drift, not by selection, is expected [92]. In other words, had the virus been allowed to spread as during normal flu/cold seasons, new SARS-CoV-2 variants with increased levels of virulence may have not been selected. 

Finally, the reason why the gamma variant emerged and found particularly fertile ground for its propagation in Brazil while failing to spread to other continents remains an open question. It may be related to the form that the pandemic was handled in the country or, more likely, to a combination of the following factors: (1) a relatively high number of individuals with metabolic dysfunctions (hypertension, obesity and others) [93]; (2) implementation of inefficient and damaging measures, such as masking of COVID-19 patients [94-95] and extensive stay-at-home mandates [96]; and (3) a random sequence of events that resulted in the emergence and positive selection of a variant of high consequence such as gamma. By its very nature, this type of study has several limitations, namely, that the hypotheses raised here cannot be easily tested and that some of the exposed criticisms have the benefit of hindsight at historical events and could have not been easily predicted. A more specific constraint was the reliance on the testing rate to obtain the number of COVID-19 cases, which in turn was used to calculate the CFR. However, this limitation is common to all studies that used the CFR metric. 

## Conclusions

The gamma variant that emerged in northern Brazil rapidly reached the state of São Paulo, becoming the prevalent variant in the first semester of 2021 and leaving a toll of tens of thousands of deaths. The gamma VOC evolved from previous variants circulating in the country as the result of a normal evolutionary rate, but its selection and fixation in the host population may have been aggravated by the physical distancing measures prevailing in Brazil at that time. Due to its high viral load, the gamma variant displayed increased transmissibility and virulence, resulting in heightened CFRs across most age tiers. Particularly, the death rate among young adults increased very significantly during this COVID-19 wave, aggravated by the deterioration in general health provoked by the stay-at-home mandate and other non-pharmaceutical interventions, which contributed, among other things, to increase weight gain and obesity in this age tier. In hindsight, a safer and more effective action would have been to allow the free spread of the virus among the young and healthy during the first wave, thus conferring total or partial immunity against more virulent variants that emerged later on.

## References

[1] (2021). WHO. Coronavirus disease (COVID-19) pandemic. https://www.who.int/emergencies/diseases/novel-coronavirus-2019.

[2] (2021). CORONAVÍRUS Brasil. https://covid.saude.gov.br.

[3] Sender R, Bar-On YM, Gleizer S, Bernshtein B, Flamholz A, Phillips R, Milo R (2021). The total number and mass of SARS-CoV-2 virions. Proc Natl Acad Sci U S A.

[4] Chaillon A, Smith DM (2021). Phylogenetic analyses of severe acute respiratory syndrome coronavirus 2 (SARS-CoV-2) B.1.1.7 lineage suggest a single origin followed by multiple exportation events versus convergent evolution. Clin Infect Dis.

[5] Tegally H, Wilkinson E, Giovanetti M (2021). Detection of a SARS-CoV-2 variant of concern in South Africa. Nature.

[6] Faria NR, Mellan TA, Whittaker C (2021). Genomics and epidemiology of the P.1 SARS-CoV-2 lineage in Manaus, Brazil. Science.

[7] M. W (2021). Genome sequencing by INSACOG shows variants of concern and a novel variant in India. Mar.

[8] Choi JY, Smith DM (2021). SARS-CoV-2 variants of concern. Yonsei Med J.

[9] Freitas AR, Beckedorff OA, Cavalcanti LP (2021). The emergence of novel SARS-CoV-2 variant P.1 in Amazonas (Brazil) was temporally associated with a change in the age and sex profile of COVID-19 mortality: a population based ecological study. Lancet Reg Health Am.

[10] Martin DP, Weaver S, Tegally H (2021). The emergence and ongoing convergent evolution of the SARS-CoV-2 N501Y lineages. Cell.

[11] Naveca FG, Nascimento V, de Souza VC (2021). COVID-19 in Amazonas, Brazil, was driven by the persistence of endemic lineages and P.1 emergence. Nat Med.

[12] Regoes RR, Nowak MA, Bonhoeffer S (2000). Evolution of virulence in a heterogeneous host population. Evolution.

[13] (2021). BBC-Brasil. Crisis in Manaus 'was inevitable, but we could have prevented collapse,' says Fiocruz scientist who suggested lockdown in September [In Portuguese]. https://www.bbc.com/portuguese/brasil-55684605.

[14] Barbosa GR, Moreira LV, Justo AF (2021). Rapid spread and high impact of the variant of concern P.1 in the largest city of Brazil. J Infect.

[15] Francisco Junior RD, Lamarca AP, de Almeida LG (2021). Turnover of SARS-CoV-2 Lineages Shaped the Pandemic and Enabled the Emergence of New Variants in the State of Rio de Janeiro, Brazil. Viruses.

[16] Zavascki AP, Vieceli T, Wink PL (2021). Advanced ventilatory support and mortality in hospitalized patients with COVID-19 caused by Gamma (P.1) variant of concern compared to other lineages: cohort study at a reference center in Brazil [Preprint]. Research Square.

[17] Martins AF, Zavascki AP, Wink PL (2021). Detection of SARS-CoV-2 lineage P.1 in patients from a region with exponentially increasing hospitalisation rate, February 2021, Rio Grande do Sul, Southern Brazil. Euro Surveill.

[18] Nonaka CK, Gräf T, Barcia CA (2021). SARS-CoV-2 variant of concern P.1 (Gamma) infection in young and middle-aged patients admitted to the intensive care units of a single hospital in Salvador, Northeast Brazil, February 2021. Int J Infect Dis.

[19] Oliveira MHS, Lippi G, Hennry BM (2021). Sudden rise in COVID-19 case fatality among young and middle-aged adults in the south of Brazil after identification of the novel b. 1.1. 28.1 (P.1) SARS-CoV-2 strain: analysis of data from the state of Parana [Preprint]. MedRxiv.

[20] Freitas ARR, Lemos DRQ, Beckedorff OB (2021). The increase in the risk of severity and fatality rate of COVID-19 in southern Brazil after the emergence of the variant of concern (VOC) SARS-CoV-2 p. 1 was greater among young adults without pre-existing risk conditions. MedRxiv [Preprint].

[21] Banho CA, Sacchetto L, Campos GR (2022). Impact of SARS-CoV-2 Gamma lineage introduction and COVID-19 vaccination on the epidemiological landscape of a Brazilian city. Commun Med (Lond).

[22] Varela AP, Prichula J, Mayer FQ (2021). SARS-CoV-2 introduction and lineage dynamics across three epidemic peaks in Southern Brazil: massive spread of P.1. Infect Genet Evol.

[23] de Souza FS, Hojo-Souza NS, da Silva CM, Guidoni DL (2021). Second wave of COVID-19 in Brazil: younger at higher risk. Eur J Epidemiol.

[24] (2021). SP against the new coronavirus [In Portuguese]. Oct.

[25] (2022). JASP, version 0.16.1 (computer software). https://jasp-stats.org/.

[26] Demombynes G (2020). COVID-19 Age-Mortality Curves Are Flatter in Developing Countries. Policy Research Working Papers. Policy Research Working Papers.

[27] Pifarré I Arolas H, Acosta E, López-Casasnovas G, Lo A, Nicodemo C, Riffe T, Myrskylä M (2021). Years of life lost to COVID-19 in 81 countries. Sci Rep.

[28] (2020). In five decades, life expectancy increases by almost 18 years in the State of São Paulo [In Portuguese]. https://www.seade.gov.br/em-cinco-decadas-esperanca-de-vida-aumenta-quase-18-anos-no-estado-de-sao-paulo/.

[29] (2021). Vaccination against Covid-19 of elderly people over 70 years old starts on March 29 in the state of SP [In Portuguese]. https://g1.globo.com/sp/sao-paulo/noticia/2021/03/15/vacinacao-contra-covid-19-de-idosos-acima-de-70-anos-comeca-em-29-de-marco-no-estado-de-sp.ghtml.

[30] (2021). VacinaJa [In Portuguese]. Vacinaja.

[31] (2021). Plano SP leitos internacoes serie nova variacao semanal [In Portuguese]. https://raw.githubusercontent.com/seade-R/dados-covid-sp/master/data/plano_sp_leitos_internacoes_serie_nova_variacao_semanal.csv.

[32] Doshi P (2020). Covid-19: Do many people have pre-existing immunity?. BMJ.

[33] Loyal L, Braun J, Henze L (2021). Cross-reactive CD4+ T cells enhance SARS-CoV-2 immune responses upon infection and vaccination. Science.

[34] Kundu R, Narean JS, Wang L (2022). Cross-reactive memory T cells associate with protection against SARS-CoV-2 infection in COVID-19 contacts. Nat Commun.

[35] Szablewski CM, Chang KT, Brown MM (2020). SARS-CoV-2 transmission and infection among attendees of an overnight camp — Georgia, June 2020. MMWR Morb Mortal Wkly Rep.

[36] Aykac K, Cura Yayla BC, Ozsurekci Y (2021). The association of viral load and disease severity in children with COVID-19. J Med Virol.

[37] Zimmermann P, Curtis N (2020). Why is COVID-19 less severe in children? A review of the proposed mechanisms underlying the age-related difference in severity of SARS-CoV-2 infections. Arch Dis Child.

[38] Levin AT, Hanage WP, Owusu-Boaitey N, Cochran KB, Walsh SP, Meyerowitz-Katz G (2020). Assessing the age specificity of infection fatality rates for COVID-19: systematic review, meta-analysis, and public policy implications. Eur J Epidemiol.

[39] Luo G, Zhang X, Zheng H, He D (2021). Infection fatality ratio and case fatality ratio of COVID-19. Int J Infect Dis.

[40] Staerk C, Wistuba T, Mayr A (2021). Estimating effective infection fatality rates during the course of the COVID-19 pandemic in Germany. BMC Public Health.

[41] Irons NJ, Raftery AE (2021). Estimating SARS-CoV-2 infections from deaths, confirmed cases, tests, and random surveys. Proc Natl Acad Sci U S A.

[42] Buss LF, Prete CA Jr, Abrahim CM (2021). Three-quarters attack rate of SARS-CoV-2 in the Brazilian Amazon during a largely unmitigated epidemic. Science.

[43] Sabino EC, Buss LF, Carvalho MP (2021). Resurgence of COVID-19 in Manaus, Brazil, despite high seroprevalence. Lancet.

[44] Hall VJ, Foulkes S, Charlett A (2021). SARS-CoV-2 infection rates of antibody-positive compared with antibody-negative health-care workers in England: a large, multicentre, prospective cohort study (SIREN). Lancet.

[45] Gazit S, Shlezinger R, Perez G (2022). SARS-CoV-2 naturally acquired immunity vs. vaccine-induced immunity, reinfections versus breakthrough infections: a retrospective cohort study. Clin Infect Dis.

[46] Abu-Raddad LJ, Chemaitelly H, Bertollini R (2021). Severity of SARS-CoV-2 reinfections as compared with primary infections. N Engl J Med.

[47] Fine P, Eames K, Heymann DL (2011). "Herd immunity": a rough guide. Clin Infect Dis.

[48] Alberca RW, Oliveira LM, Branco AC, Pereira NZ, Sato MN (2021). Obesity as a risk factor for COVID-19: an overview. Crit Rev Food Sci Nutr.

[49] Sanchis-Gomar F, Lavie CJ, Mehra MR, Henry BM, Lippi G (2020). Obesity and outcomes in COVID- 19: when an epidemic and pandemic collide. Mayo Clin Proc.

[50] Ruíz-Roso MB, de Carvalho Padilha P, Matilla-Escalante DC (2020). Changes of physical activity and ultra-processed food consumption in adolescents from different countries during COVID-19 pandemic: an observational study. Nutrients.

[51] Stockwell S, Trott M, Tully M (2021). Changes in physical activity and sedentary behaviours from before to during the COVID-19 pandemic lockdown: a systematic review. BMJ Open Sport Exerc Med.

[52] Lange SJ, Kompaniyets L, Freedman DS, Kraus EM, Porter R, Blanck HM, Goodman AB (2021). Longitudinal Trends in body mass index before and during the COVID-19 pandemic among persons aged 2-19 years - United States, 2018-2020. MMWR Morb Mortal Wkly Rep.

[53] Woolford SJ, Sidell M, Li X, Else V, Young DR, Resnicow K, Koebnick C (2021). Changes in body mass index among children and adolescents during the COVID-19 pandemic. JAMA.

[54] Sophie Bethune (2021). One year on: unhealthy weight gains, increased drinking reported by Americans coping with pandemic stress. APA.

[55] Robinson E, Boyland E, Chisholm A (2021). Obesity, eating behavior and physical activity during COVID-19 lockdown: a study of UK adults. Appetite.

[56] Cava E, Neri B, Carbonelli MG, Riso S, Carbone S (2021). Obesity pandemic during COVID-19 outbreak: narrative review and future considerations. Clin Nutr.

[57] Shrock E, Fujimura E, Kula T (2020). Viral epitope profiling of COVID-19 patients reveals cross-reactivity and correlates of severity. Science.

[58] Chiu WT, Scholl J, Li YJ, Wu J (2021). So few COVID-19 cases in Taiwan: has population immune health played a role?. Front Public Health.

[59] Yaqinuddin A (2020). Cross-immunity between respiratory coronaviruses may limit COVID-19 fatalities. Med Hypotheses.

[60] Dijkstra JM, Frenette AP, Dixon B (2021). Most Japanese individuals are genetically predisposed to recognize an immunogenic protein fragment shared between COVID-19 and common cold coronaviruses. F1000Res.

[61] Vimaleswaran KS, Forouhi NG, Khunti K (2021). Vitamin D and COVID-19. BMJ.

[62] Carpagnano GE, Di Lecce V, Quaranta VN (2021). Vitamin D deficiency as a predictor of poor prognosis in patients with acute respiratory failure due to COVID-19. J Endocrinol Invest.

[63] Pereira M, Dantas Damascena A, Galvão Azevedo LM, de Almeida Oliveira T, da Mota Santana J (2022). Vitamin D deficiency aggravates COVID-19: systematic review and meta-analysis. Crit Rev Food Sci Nutr.

[64] da Silveira MP, da Silva Fagundes KK, Bizuti MR, Starck É, Rossi RC, de Resende E Silva DT (2021). Physical exercise as a tool to help the immune system against COVID-19: an integrative review of the current literature. Clin Exp Med.

[65] Simpson RJ, Kunz H, Agha N, Graff R (2015). Exercise and the regulation of immune functions. Prog Mol Biol Transl Sci.

[66] Laddu DR, Lavie CJ, Phillips SA, Arena R (2021). Physical activity for immunity protection: inoculating populations with healthy living medicine in preparation for the next pandemic. Prog Cardiovasc Dis.

[67] Simonsen L, Chowell G, Andreasen V, Gaffey R, Barry J, Olson D, Viboud C (2018). A review of the 1918 herald pandemic wave: importance for contemporary pandemic response strategies. Ann Epidemiol.

[68] Barry JM, Viboud C, Simonsen L (2008). Cross-protection between successive waves of the 1918-1919 influenza pandemic: epidemiological evidence from US Army camps and from Britain. J Infect Dis.

[69] Shanks GD, Mackenzie A, McLaughlin R, Waller M, Dennis P, Lee SE, Brundage JF (2010). Mortality risk factors during the 1918-1919 influenza pandemic in the Australian army. J Infect Dis.

[70] M. Kulldorff, S. Gupta, and J (2020). Great Barrington Declaration. https://gbdeclaration.org.

[71] Akamatsu T, Nagae T, Osawa M, Satsukawa K, Sakai T, Mizutani D (2021). Model-based analysis on social acceptability and feasibility of a focused protection strategy against the COVID-19 pandemic. Sci Rep.

[72] Boni MF, Nguyen TD, de Jong MD, van Doorn HR (2013). Virulence attenuation during an influenza A/H5N1 pandemic. Philos Trans R Soc Lond B Biol Sci.

[73] Simon-Loriere E, Holmes EC (2011). Why do RNA viruses recombine?. Nat Rev Microbiol.

[74] Shapshak P, Chiappelli F, Somboonwit C, Sinnott J (2011). The influenza pandemic of 2009: lessons and implications. Mol Diagn Ther.

[75] T Day (2003). Virulence evolution and the timing of disease life-history events. Trends Ecol Evol.

[76] Mackinnon MJ, Read AF (1999). Genetic relationships between parasite virulence and transmission in the rodent malaria Plasmodium chabaudi. Evolution.

[77] de Roode JC, Yates AJ, Altizer S (2008). Virulence-transmission trade-offs and population divergence in virulence in a naturally occurring butterfly parasite. Proc Natl Acad Sci U S A.

[78] de Jong MD, Simmons CP, Thanh TT (2006). Fatal outcome of human influenza A (H5N1) is associated with high viral load and hypercytokinemia. Nat Med.

[79] Makov-Assif M, Krispin S, Ben-Shlomo Y, Holander T, Dagan N, Balicer R, Barda N (2022). The association between real-time reverse transcriptase polymerase chain reaction cycle threshold values, symptoms and disease severity among COVID-19 patients in the community: a retrospective cohort study. Infect Dis (Lond).

[80] Knudtzen FC, Jensen TG, Lindvig SO (2021). SARS-CoV-2 viral load as a predictor for disease severity in outpatients and hospitalised patients with COVID-19: a prospective cohort study. PLoS One.

[81] Singh V, Agarwal J, Garg J, Saqui̇b M, Das A, Sen M (2021). Role of cycle threshold of RT-PCR in the prediction of COVID-19 cases. J Microbiol Infect Dis.

[82] Wright J, Achana F, Diwakar L (2021). Cycle threshold values are inversely associated with poorer outcomes in hospitalized patients with COVID-19: a prospective, observational cohort study conducted at a UK tertiary hospital. Int J Infect Dis.

[83] Rabaan AA, Tirupathi R, Sule AA (2021). Viral dynamics and real-time RT-PCR ct values correlation with disease severity in COVID-19. Diagnostics (Basel).

[84] Shenoy S (2021). SARS-CoV-2 (COVID-19), viral load and clinical outcomes; lessons learned one year into the pandemic: a systematic review. World J Crit Care Med.

[85] Kawasuji H, Morinaga Y, Tani H (2022). SARS-CoV-2 RNAemia with a higher nasopharyngeal viral load is strongly associated with disease severity and mortality in patients with COVID-19. J Med Virol.

[86] Bjorkman KK, Saldi TK, Lasda E (2021). Higher viral load drives infrequent severe acute respiratory syndrome coronavirus 2 transmission between asymptomatic residence hall roommates. J Infect Dis.

[87] Kawasuji H, Takegoshi Y, Kaneda M (2020). Transmissibility of COVID-19 depends on the viral load around onset in adult and symptomatic patients. PLoS One.

[88] He X, Lau EH, Wu P (2020). Temporal dynamics in viral shedding and transmissibility of COVID-19. Nat Med.

[89] Marks M, Millat-Martinez P, Ouchi D (2021). Transmission of COVID-19 in 282 clusters in Catalonia, Spain: a cohort study. Lancet Infect Dis.

[90] Yang Q, Saldi TK, Gonzales PK (2021). Just 2% of SARS-CoV-2-positive individuals carry 90% of the virus circulating in communities. Proc Natl Acad Sci U S A.

[91] González-Candelas F, Shaw MA, Phan T (2021). One year into the pandemic: short-term evolution of SARS-CoV-2 and emergence of new lineages. Infect Genet Evol.

[92] MacLean OA, Lytras S, Weaver S (2021). Natural selection in the evolution of SARS-CoV-2 in bats created a generalist virus and highly capable human pathogen. PLoS Biol.

[93] Cadegiani FA, Wambier CG, Goren A (2020). Spironolactone: an anti-androgenic and anti-hypertensive drug that may provide protection against the novel coronavirus (SARS-CoV-2) induced acute respiratory distress syndrome (ARDS) in COVID-19. Front Med (Lausanne).

[94] Fögen Z (2022). The Foegen effect: a mechanism by which facemasks contribute to the COVID-19 case fatality rate. Medicine (Baltimore).

[95] Spira B (2022). Correlation between mask compliance and COVID-19 outcomes in Europe. Cureus.

[96] Herby J, Jonung L, Hanke SH (2022). A literature review and meta-analysis of the effects of lockdowns on COVID-19 mortality. Studies in Applied Economics.

